# Development of multivalent nanobodies blocking SARS-CoV-2 infection by targeting RBD of spike protein

**DOI:** 10.1186/s12951-021-00768-w

**Published:** 2021-01-29

**Authors:** Qizhong Lu, Zongliang Zhang, Hexian Li, Kunhong Zhong, Qin Zhao, Zeng Wang, Zhiguo Wu, Donghui Yang, Shuang Sun, Nian Yang, Meijun Zheng, Qiang Chen, Cheng Long, Wenhao Guo, Hui Yang, Chunlai Nie, Aiping Tong

**Affiliations:** 1 State Key Laboratory of Biotherapy and Cancer Center, West China Hospital, Sichuan University, Chengdu, 610041 China; 2 Department of Preventive Veterinary Medicine, College of Veterinary Medicine, Northwest A&F University, Yangling, 712100 China; 3 College of Veterinary Medicine, Shaanxi Center of Stem Cells Engineering and Technology, Northwest A&F University, Yangling, 712100 China; 4 Department of Otolaryngology, Head and Neck Surgery, West China Hospital, Sichuan University, Chengdu, 610041 China; 5 Department of Orthopaedics, West China Hospital, Sichuan University, Chengdu, 610041 China

**Keywords:** SARS-CoV-2, Nanobody, Spike, RBD, Pseudovirus neutralization

## Abstract

**Background:**

The outbreak and pandemic of coronavirus SARS-CoV-2 caused significant threaten to global public health and economic consequences. It is extremely urgent that global people must take actions to develop safe and effective preventions and therapeutics. Nanobodies, which are derived from single‑chain camelid antibodies, had shown antiviral properties in various challenge viruses. In this study, multivalent nanobodies with high affinity blocking SARS-CoV-2 spike interaction with ACE2 protein were developed.

**Results:**

Totally, four specific nanobodies against spike protein and its RBD domain were screened from a naïve VHH library. Among them, Nb91-hFc and Nb3-hFc demonstrated antiviral activity by neutralizing spike pseudotyped viruses in vitro. Subsequently, multivalent nanobodies were constructed to improve the neutralizing capacity. As a result, heterodimer nanobody Nb91-Nb3-hFc exhibited the strongest RBD-binding affinity and neutralizing ability against SARS-CoV-2 pseudoviruses with an IC_50_ value at approximately 1.54 nM.

**Conclusions:**

The present study indicated that naïve VHH library could be used as a potential resource for rapid acquisition and exploitation of antiviral nanobodies. Heterodimer nanobody Nb91-Nb3-hFc may serve as a potential therapeutic agent for the treatment of COVID-19.
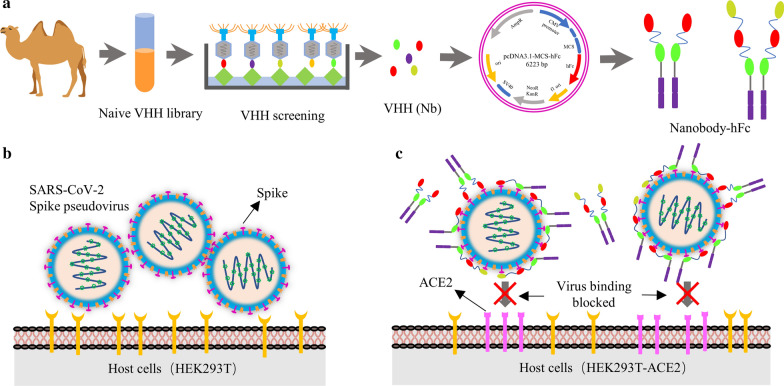

## Background

Coronavirus disease 2019 (COVID-19) is caused by infection of severe acute respiratory syndrome coronavirus 2 (SARS-CoV-2) [1, 2]. It has spread global and had been announced by World Health Organization (WHO) in March 2020 as the first coronavirus severe pandemic in the history of humanity [3, 4]. More than 83.92 million individuals have been infected and caused about 1.82 million people deaths globally (as of January 2, 2021, source: Johns Hopkins University), and the number is still increasing. It is extremely urgent that global people must take actions to develop safe and effective preventions and therapeutics.

SARS-CoV-2 is an enveloped virus that belongs to the family *Coronaviridae*, the subfamily *Orthocoronaviridae* and genus *β-coronavirus* [5]. The virus genome is a positive-sense, single-stranded RNA with a full length of 30.0 kb, which is 96.2 % identical to a bat CoV RaTG13, whereas it shares 79.6 % identity to SARS-CoV [5, 6]. Its genome consists of six functional open-reading frames (ORFs), which encoded replicase (ORF1a/ORF1b), membrane (M), spike glycoprotein (S), envelope (E) and nucleocapsid (N), most of the proteins encoded by SARS-CoV-2 are similar with SARS-CoV [7, 8]. S protein can form a homotrimer complex and extrude from envelope to form the coronal in terms of morphological structure. It can be structurally or functionally divided into two subunits, called S1 and S2. S1 subunit contains the receptor-binding domain (RBD), which binds to the extracellular domain of receptor angiotensin converting enzyme 2 (ACE2) and mediate the virus entry into host cells, while the S2 subunit is necessary for membrane fusion [9–12]. Based on the characteristics of SARS-CoV-2 RBD immunogen could induce neutralizing antibody in animals and is necessary for virus infection in host cells, thus, it can be used as a good target for the development of neutralizing antibodies [13, 14].

Heavy-chain only antibodies (hcAbs) derived from camelids or sharks that devoid of light chains and lack CH1, nevertheless have an extensive antigen-binding repertoire, its variable domain was named VHH or nanobody (Nb) (15 kDa) [15–18]. Nanobody offer advantages including high affinity and specificity, smaller size (1/10th the size of conventional monoclonal antibodies), thermostability, low immunogenicity, and excellent tissue penetration-characteristics that are widely applied in oncotherapy, diagnosis and monitoring of disease, and prevent virus infection [19–24]. For example, numerous studies about Nbs antiviral activity for various challenging viruses have been reported, including MERS, HIV, HCV, IAV and SFTSV [24–27]. While many candidates are in preclinical development and several antibodies (VIR-7831, LY-CoV016, BGB-DXP593, and CT-P59) have entered late-stage clinical trials, two neutralizing antibodies, Lilly’s LY-Cov555 and Regeneron’s REGN-COV2, have received FDA emergency use authorization for the treatment of COVID-19. For nanobodies, most of them are in preclinical trials. For example, Twist Bioscience Corporation recently announced two nanobodies, TB202-3 and TB202-63, protect against weight loss, a key indicator of disease severity, at the dose of 1 mg/kg in a preclinical hamster challenge model.

**Scheme 1:**
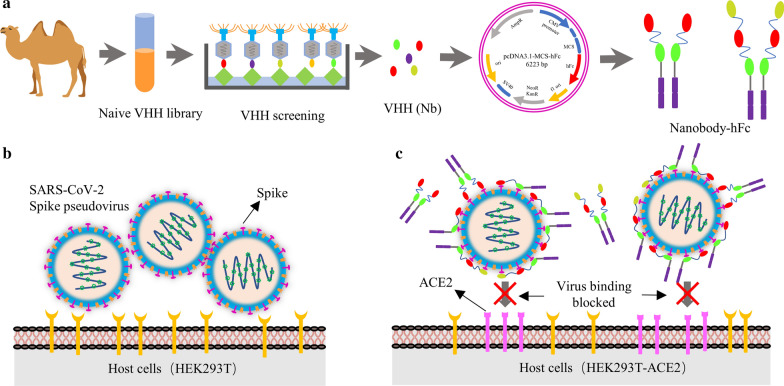
Schematic presentation of screening nanobodies, SARS-CoV-2 spike pseudovirus production and neutralization assay. **a** Screening specific nanobodies against S and RBD protein from naïve VHH library, and the expression of nanobody-hFc fusions. **b** Production of SARS-CoV-2 spike pseudovirus using HEK293T cells as the host cells. **c** Determination the neutralizing activity of multivalent nanobodies

Here, several nanobodies directed to spike protein and its RBD domain with high affinity were obtained after multiple rounds of enrichment from a naïve VHH library (Scheme 1a). Based on the production platform of the nanobody-hFc, S and RBD protein specific nanobodies were expressed (Scheme 1a). To determinate the neutralizing activity of specific nanobodies, the SARS-CoV-2 spike pseudotyped lentivirus were produced firstly using HEK293T cells (Scheme 1b). Neutralizing nanobodies could significantly inhibit SARS-CoV-2 pseudoviruses infection in host HEK293T-ACE2 cells through blocking spike protein interaction with ACE2 by targeting RBD (Scheme 1c). We believe that nanobodies may serve as a potential agent for prevention and therapy of COVID-19.

## Materials and methods

### Cell lines and vectors

HEK293T cell lines were purchased from ATCC and cultured in Dulbecco’s Modified Eagle’s Medium (Gibco, USA) containing 10 % fetal bovine serum (FBS, BI, USA) at 37 °C in 5 % CO_2_. Spodoptera fruited (sf9) cells were maintained in the SIM SF medium (Sino Biological, Beijing, China). All cell lines have been tested negative for contamination with mycoplasma. HEK293T cells were used to construct HEK293T-ACE2 stable cell lines, express recombinant nanobodies, and produce SARS-CoV-2 pseudovirus of the novel coronavirus; sf9 cells were applied to express spike and RBD protein of SARS-CoV-2. The pMECS vector was used to construct phage display library. The pcDNA3.1 vector (V790-20, invitrogen, USA) was used for eukaryotic expression of the nanobodies. pLenti-EGFP-luciferase expressing plasmid and gag/pol plasmid were used to prepare SARS-CoV-2 pseudoviruses.

### Gene cloning, protein expression and purification of recombinant SARS-CoV-2 proteins

The spike and RBD protein of SARS-CoV-2 were expressed using the Bac-to-Bac baculovirus expression system (Invitrogen) as previously described [14]. Briefly, a gp67 signal peptide sequence [28] was inserted into pFastBac1 vector between *Bam*HI and *Eco*RI restriction sites, and then the coding sequence (codon optimized for insect cells) of spike ECD (extracellular domain, 16–1213 aa) and RBD (319–545 aa) of SARS-CoV-2 Wuhan-Hu-1 isolate (Genbank accession number MN908947) were followed by the gp67 signal peptide. In addition, 8× His tag was fused to C-terminal to profit protein purification. The bacmid was transfected into sf9 cells with LipoInsect Transfection regent (Beyotime Biotechnology, Jiangsu, China) according to the manufacture’s instruction for recombinant baculovirus package and protein expression. Subsequently, the culture supernatant that containing S and RBD protein were harvested after 72 h transfection and purified using Ni-NTA 6FF Agarose (SMART, Changzhou, China), respectively. The expression and purity of recombinant were analyzed by SDS-PAGE.

### Construction of naïve VHH library

Total 200 mL Bactrian camel blood samples (containing 50 camels) were collected from Jinchang city, Gansu province in China. The peripheral blood lymphocytes (PBLs) were extracted by Leucosep® tubes (Greiner Bio-One, Germany) for naïve library construction. Total RNA was extracted and used to synthesise the cDNA. Next, the VHH genes were amplified by nest-PCR according to previous description [29–31], and then cloned into phagemid pMECS vector. The recombinant phagemids were electro-transformed into freshly competent *E. coli* TG1 Electrocompetent cells. Cells were cultured on LB agar plates that containing ampicillin and D-glucose overnight at 37 °C. On the second day, the cells were scraped and stored at − 80 °C at LB medium, and then the positive rate of the constructed library was determined according to previous description [32]. Finally, 36 clones were randomly selected for sequencing to analyze the library’s diversity.

### Screening and identification specific nanobodies against SARS-CoV-2 spike and RBD protein

To select the spike and RBD nanobodies, four rounds of phage rescue and screening were performed as described previously [30, 31, 33]. Briefly, purified S and RBD protein were immobilized in microtiter plate (Nunc, Thermo). For each round an uncoated well was used as a negative control. The wells were washed with PBS’T buffer (PBS with 0.05 % Tween 20 (v/v)) and then were blocked with blocking buffer (PBS’T containing 5 % (w/v) skimmed milk) at 37 °C for 1 h. Then, rescued recombinant phages were added to microplate wells that containing S and RBD protein and incubated for 1 h at 37 °C. After being washed, the retained phages were eluted with Glycine-HCl buffer (pH 3.0) and neutralized immediately with Tris-HCl buffer (pH 8.5) to a neutral condition. Next, the eluted phages were used to infect *E. coli* TG1 cells and amplified overnight at 37 °C after infecting with M13KO7 helper phages. Subsequently, the amplified phages were purified using PEG 4,000/NaCl precipitation for the next round bio-panning.

After four rounds of screening, the enrichment of S and RBD specific phage particles were calculated with polyclonal phage ELISA, and then 96 clones were picked randomly for monoclonal identification using an indirect ELISA with HRP-conjugated goat anti-M13 IgG antibody (Sino Biological, Beijing, China). Finally, all positive clones were sequenced and grouped based on their complementary determining regions (CDRs) amino acid sequence.

### VHH cloning into HEK293FT expression vector

A pcDNA3.1 expression vector was constructed firstly to express the S and RBD protein specific nanobodies in HEK293FT cells. In brief, some elements were designed and cloned into commercial pcDNA3.1(+) vector between *Nhe*I and *Xba*I sites. DNA sequences, including a secreting signal sequence from the human IgGκ chain, multiple cloning site (MCS), human IgG1 Fc, and 6× His tag following a stop coding sequence, were synthesized (GENERAL BIOL, Anhui, China) and cloned into the multiple cloning sites of the commercial vector pcDNA3.1, and named as pcDNA3.1-MCS-hFc. The VHHs encoding sequences were amplified by PCR using the Nbs-F, Nbs-R primers (Table 1) and cloned between the *Bam*HI and *Xho*I sites in the pcDNA3.1-MCS-hFc vector. The positive recombinant plasmids were confirmed by sequencing.

**Table 1:** Primer sequences of amplification VHH genes for recombinant plasmids pcDNA3.1-Nbs-hFc construction

Primer	Sequences
Nbs-F	5′-CAGGGATCCCAGGTGCAGCTGGTGGAGTC-3′
Nbs-R	5′-CGCCTCGAGTGAGGAGACGGTGACCTGGG-3′
biNbs-R1	5′-TGAACCGCCTCCACCGCTGCCGCCTCCGCCTGAGGAGACGGTGACCTGGG-3′
biNbs-F2	5′-GGTGGAGGCGGTTCAGGAGGTGGCGGATCTCAGGTGCAGCTGGTGGAGTC-3′
triNbs-R32	5′-AGATCCGCCACCTCCGCTGCCGCCTCCGCCTGAGGAGACGGTGACCTGGG-3′
triNbs-F3	5′-GGAGGTGGCGGATCTGGTGGAGGCGGTTCACAGGTGCAGCTGGTGGAGTC-3′

Underline indicates position of restriction enzyme sites

### Generating bivalent and trivalent VHHs for HEK293FT expression

To generate bivalent and trivalent tail-to-head VHH constructs, the VHH sequences were amplified through combination the following primers, Nbs-F, Nbs-R, biNbs-R1, biNbs-F2, triNbs-R32, triNbs-F3 (Table 1), digested with both *Bam*HI and *Xho*I enzymes and ligated into the vector pcDNA3.1-MCS-hFc. The positive plasmids were propagated with *E. coli* TransStbl3 cells and used to express recombinant VHH protein by transfection HEK293FT cells.

### Expression and purification of nanobodies

To produce nanobody-hFc recombinant fusion proteins, the vectors containing monovalent, bivalent and trivalent VHHs were transfected into mammalian cell line HEK293FT cells cultured in Freestyle medium (Gibco, USA) using polyetherimide reagent (PEI, Polysciences Inc. Warrington, USA) based on the manufacture’s instruction. After 5 days, the medium that containing secreted nanobody-hFc fusion proteins were harvested by centrifugation at 10,000×*g* for 20 min at 4 °C, and then incubated with Ni-NTA 6FF Agarose for purification. The nanobody-hFc recombinant fusion proteins were eluted using elution buffer (20 mM Tris, 250 mM NaCl, 250 mM imidazole, pH 7.8). The expression and purification were verified using SDS-PAGE and subsequent Coomassie Blue staining. Next, purified VHHs were concentrated on filter tubes (Milipore, USA) and the elution buffer containing imidazole was exchanged with PBS (pH 7.4). Finally, purified VHHs were used directly or stored at − 20 °C.

### Indirect ELISA assay

Determination the binding of nanobody-hFc fusion proteins against S and RBD protein, microtiter plates were coated overnight at 4 °C with recombinant S and RBD protein, respectively. After washed three times with PBS’T, the coated plates were blocked with 5 % skimmed milk in PBS’T. Dilution series (from 10^2^ to 10^− 5^ µg/mL) of nanobody-hFc fusion proteins were added to the wells, followed by adding HRP-conjugated goat anti-human IgG (1/2000, Beyotime Biotechnology). After washing, tetramethylbenzidine liquid substrate (TMB, Sigma) was added to the plates and the reaction was stopped with 2 M H_2_SO_4_, and the optical density at 450 nm (OD_450nm_) was measured using an automatic ELISA microplate reader. The binding ability was determined using four-parameter nonlinear regression curve fit (Graphpad Prism 5.0).

### Production of pseudoviruses

SARS-CoV-2 S pseudovirus production system was developed in our laboratory as previously described [14]. Briefly, a pLenti-EGFP-luciferase expressing plasmid, a plasmid encoding codon optimized SARS-CoV-2 S protein and a gag/pol expression plasmid were co-transfected into HEK293T cells using polyetherimide reagent (PEI). After transfection 6 h, the medium was replaced with fresh DMEM medium supplemented with 10 % FBS. 48 h post-transfection, the supernatant was harvested that containing SARS-CoV-2 pseudovirus, and the supernatant was filtered through 0.45 µm-pore cellulose acetate membranes. To increase the titer of pseudovirus, the supernatants were concentrated to 1 mL by ultracentrifugation and stored at − 80 °C until use.

### Pseudotyped virus neutralization assay

To determinate the neutralizing activity of SARS-CoV-2 spike and RBD-specific nanobodies against SARS-CoV-2 infection, a pseudovirus neutralization assay was performed. HEK293T-ACE2 stable cell lines were constructed as previously described [14]. In brief, HEK293T cells were transfected with a lentivirus vector encoding human ACE2 and puromycin selection marker. Cells were selected with puromycin, and puromycin-resistant clones were expanded and verified by FACS for ACE2 expression. HEK293T-ACE2 cells were plated into 96-well cell-culture plates with 2 × 10^4^ cells/well and cultured overnight at 37 °C with 5 % CO_2_. The pseudovirus was preincubated with tenfold serial dilution of Nbs at 37 °C for 1 h before being added to cells. Pseudovirus in culture media without Nbs were used as negative control. Medium were changed the following day after infection. 48 h later, EGFP expression was determined by fluorescent microscopy and flow cytometry to evaluate the neutralization ability. While the cells were lysed using lysis reagent (Promega) after washing with PBS, and relative luciferase activity was measured immediately in the Ultra 384 luminometer (Tecan). The relative luminescence signals (RLU) from the negative control wells were normalized and used to calculate neutralization percentage for each concentration.

### Statistical analysis

All statistical analyses were performed using GraphPad Prism version 5.0 (GraphPad Software, San Diego, CA, USA). All presented data were shown as mean ± SD, which contains three replicates. Comparisons among multiple groups were performed using a two-way ANOVA test with Bonferroni post-test. *P*-values < 0.05 were considered statistically significant (*P < 0.05; **P < 0.01; ***P < 0.001).

## Results

### Construction of the naïve VHH library

**Fig. 1:**
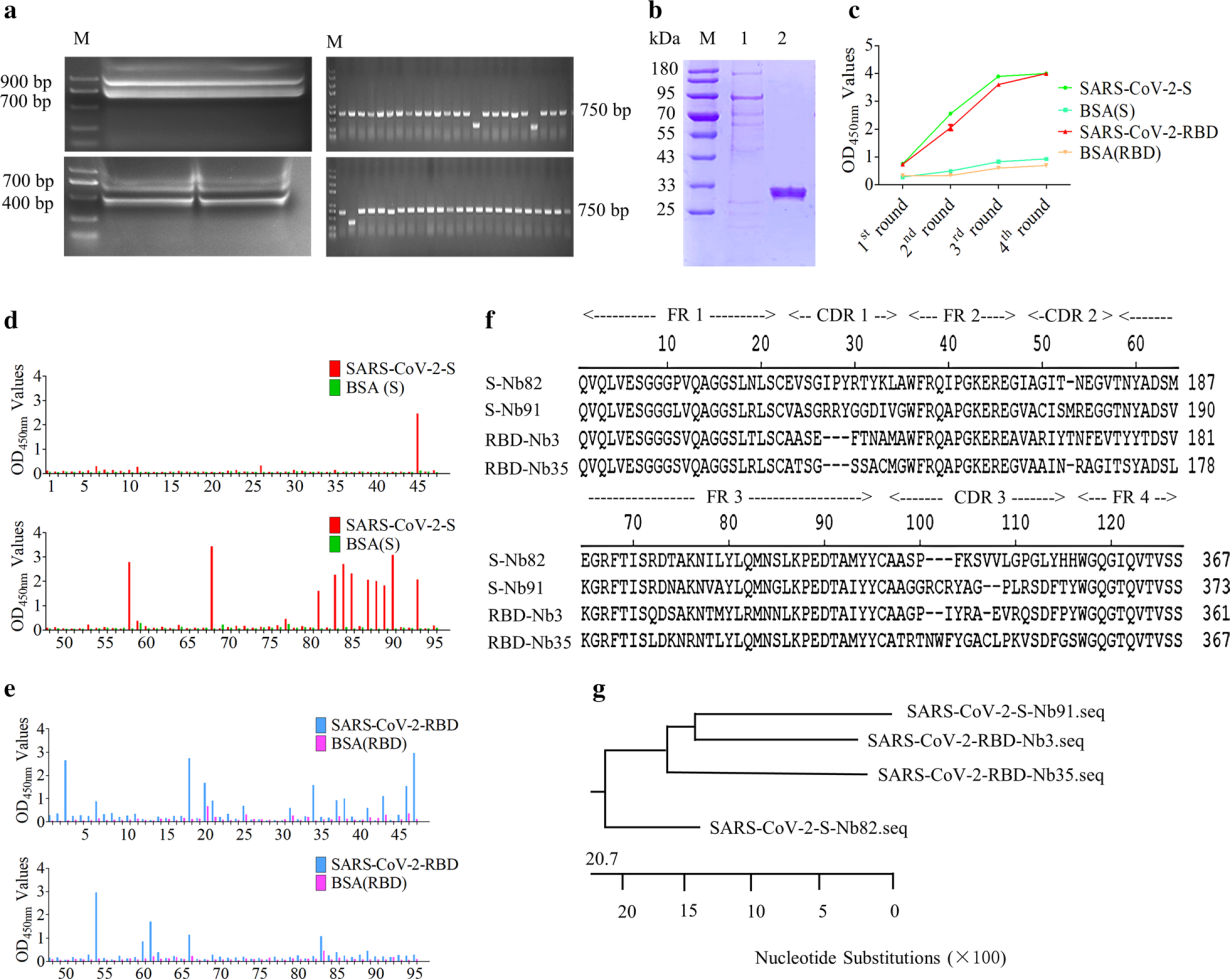
A naïve VHH library construction and screening the SARS-CoV-2 S and RBD specific nanobodies. **a** Amplification of VHH genes (~ 400 bp) by nest-PCR from PBLs, and estimation the correct insertion rate of VHH library. **b** Expression and purification of recombinant S and RBD protein were detected using SDS-PAGE. M: Marker; lane 1: purified S protein; lane 2: purified RBD protein. **c** Analysis of the enrichment of phage particles against S and RBD specific nanobodies with polyclonal indirect ELISA. **d** and **e** Identification of the recombinant VHH-gpIII protein from the 96 clones specifically binding with S protein (17 clones) and its RBD domain (40 clones), respectively. **f** Alignment of the amino acid sequences of specific nanobodies against S and RBD protein. The sequences were classified into four groups according to their CDRs. **g** Phylogenetic tree of the isolated S and RBD-directed VHHs based on the neighbor joining method by using DNASTAR software

Total 4.3 × 10^8^ PBLs were isolated from 200 mL Bactrian camel blood samples. After reverse-transcription using the extracted RNA as a template, a target band of about 400 bp in size was amplified by nest-PCR (Fig. 1a), and then the PCR fragments and pMECS vectors were digested, ligated, and transformed into TG1 cells. Finally, a phage display naïve VHH library was successfully constructed that containing of approximately 5.7 × 10^9^ individual transformants. Additionally, the insertion rate of VHH genes were evaluated by PCR through randomly picked 48 clones, which was determined to be 93.75 % (Fig. 1a). Subsequently, the sequences of 36 individual clones indicated that the library had good diversity (data not shown).

### Preparation of the SARS-CoV-2 recombinant proteins

The recombinant spike (ECD) protein and its RBD domain of SARS-CoV-2 were produced using Baculovirus Expression System. The culture supernatant that containing recombinant S and RBD proteins was harvested and purified by immobilized metal affinity chromatography (IMAC) using Ni-NTA. The results showed that recombinant RBD proteins (27 kDa) were expressed successfully and the target was obtained after purification (Fig. 1b), however, there were several bands in the S protein lane (Fig. 1b), and the possible reason is that S protein was cleaved at the S1/S2 protease cleavage site, in keeping with published data [34–36].

### Screening and identification of specific nanobodies against S and RBD protein

After four rounds bio-panning using purified S and RBD protein, the specific nanobodies particles against S and RBD protein were enriched (Fig. 1c) and the ratio of positive/negative clones (P/N) increased, from 17.8 to 1.5 × 10^3^, and from 1.5 to 1.8 × 10^3^, respectively (Table 2). The indirect ELISA results revealed that 17 clones (Fig. 1d), and 40 clones (Fig. 1e) could specifically bind with S and RBD protein, respectively. After the positive clones were sequenced, 4 unique nanobodies were obtained according to the amino acid sequences of the CDRs (Fig. 1f). Multiple sequence alignment and phylogenetic analysis using the neighbor-joining method revealed that 2 unique S-Nbs and 2 unique RBD-Nbs were isolated. The gene evolution and homology between Nb91-hFc and -Nb3-hFc were higher than others (Fig. 1g).

**Table 2:** Enrichment of phage particles against SARS-CoV-2-spike and -RBD specific nanobodies during four rounds of panning

Round of screening	Input (pfu/well)	P output (pfu/well)		N output (pfu/well)		Recovery (P/input)		P/N	
		S	RBD	S	RBD	S	RBD	S	RBD
1st round	1.0 × 10^11^	1.5 × 10^4^	1.4 × 10^4^	8.4 × 10^3^	9.1 × 10^3^	1.5 × 10^− 7^	1.4 × 10^− 7^	17.8	1.5
2nd round	1.0 × 10^11^	6.5 × 10^5^	3.4 × 10^5^	3.4 × 10^3^	2.9 × 10^3^	6.5 × 10^− 6^	3.4 × 10^− 6^	1.9 × 10^2^	1.2 × 10^2^
3rd round	1.0 × 10^11^	2.6 × 10^7^	1.9 × 10^7^	4.5 × 10^4^	3.8 × 10^4^	2.6 × 10^− 4^	1.9 × 10^− 4^	5.7 × 10^2^	5.0 × 10^2^
4th round	1.0 × 10^11^	5.4 × 10^8^	5.2 × 10^8^	3.6 × 10^5^	2.7 × 10^5^	5.4 × 10^− 2^	5.2 × 10^− 2^	1.5 × 10^3^	1.8 × 10^3^

### Expression and purification of the nanobodies against S and RBD protein using HEK293FT mammalian system

**Fig. 2:**
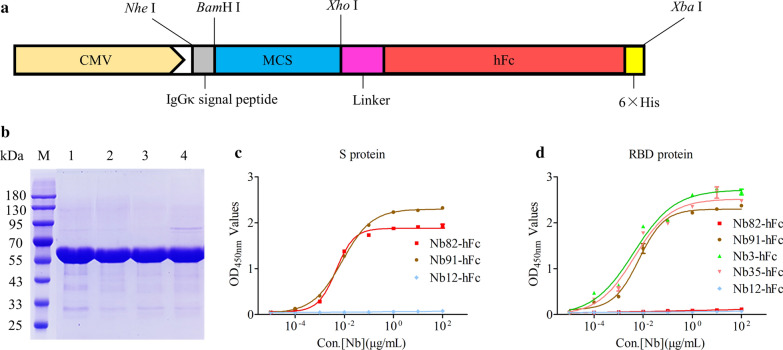
Expression, purification and binding ability determination of monovalent nanobodies. **a** Schematic presentation of the vector for nanobody expression in mammalian cells. **b** SDS-PAGE detection of the expression and purification of recombinant nanobody-hFc fusions (55 kDa). M: Marker; lane 1: Nb82-hFc; lane 2: Nb91-hFc; lane 3: Nb3-hFc; lane 4: Nb35-hFc. **c** Measurement the binding ability of recombinant monovalent nanobodies using indirect ELISA

**Fig. 3:**
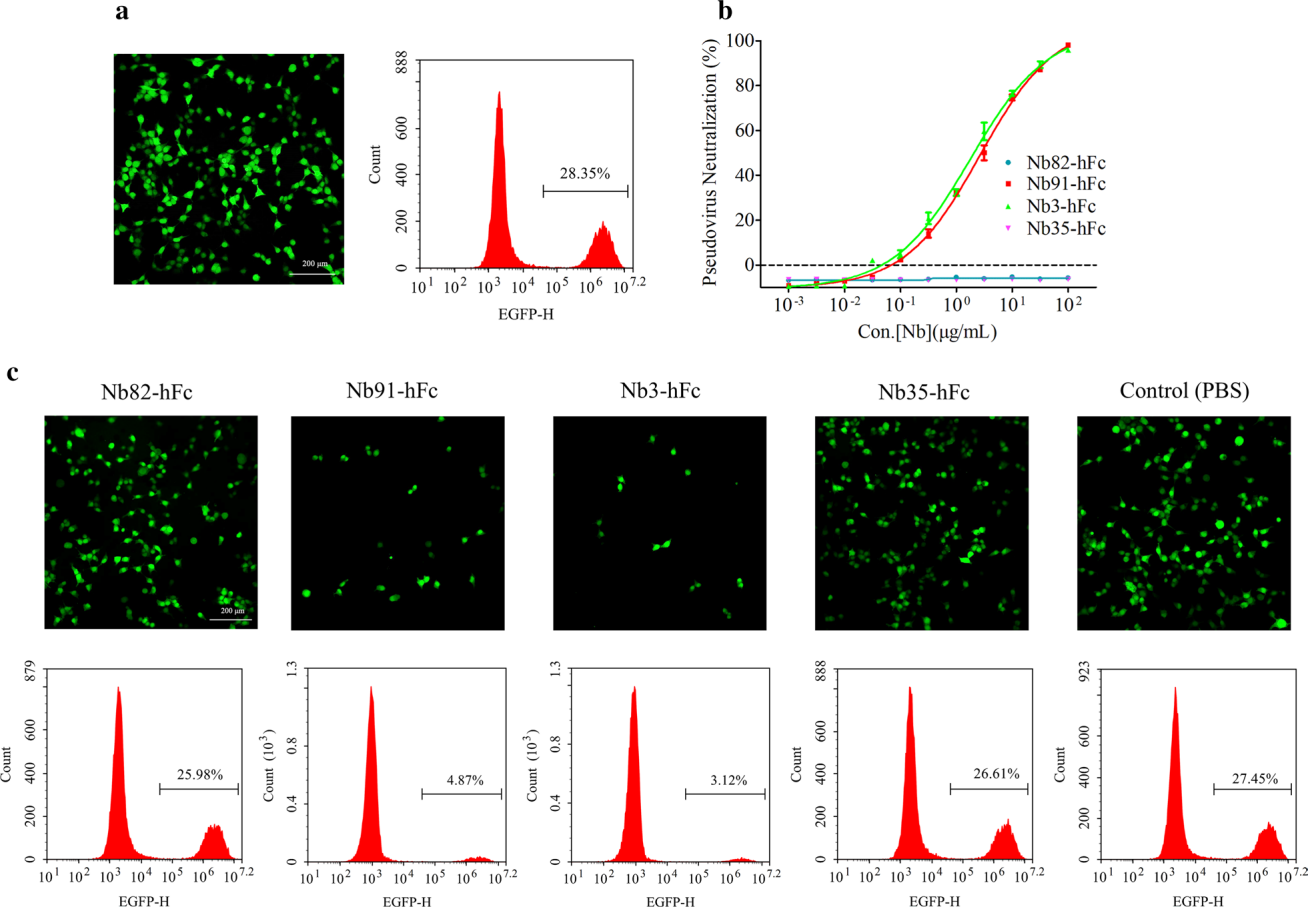
The selection of the potent neutralizing nanobodies. **a** Analysis the infection efficiency of pseudovirus with fluorescent microscopy and flow cytometry assays. **b** and **c** Measurement the neutralization potency of nanobodies with luciferase assay, fluorescent microscopy and flow cytometry assays

The platform of expression nanobody-hFc fusion proteins were constructed successfully based on the commercial pcDNA3.1 vector. In detail, the DNA sequences, including a IgGκ singal peptide, MCS, human IgG1 Fc, 6× His tag, and stop codon were cloned into pcDNA3.1 vector using *Nhe*I and *Xba*I enzymes digestion (Fig. 2a). In addition, a flexible linker was added between Nbs and human IgG1 Fc to increase the flexibility and avoid the formation of higher structures that affect the function of Nbs (Fig. 2a). SDS-PAGE results showed that high purity Nbs-hFc fusions (Nb82-hFc, Nb91-hFc, Nb3-hFc, and Nb35-hFc) were obtained after purification (Fig. 2b). The indirect ELISA results revealed that: (1) Nb82-hFc could specifically bind with S protein (Fig. 2c), but did not bind with RBD protein (Fig. 2d); (2) Nb91-hFc could bind with S protein (Fig. 2c), as well as RBD protein (Fig. 2d); (3) Nb3-hFc and Nb35-hFc could recognize RBD protein (Fig. 2d); Nb12-hFc targeting VP2 protein of porcine parvovirus was used as the negative control.

### Nanododies neutralize SARS-CoV-2 pseudotyped viruses

To evaluate the antiviral activity of the S and RBD-directed nanobodies, SARS-CoV-2 S pseudovirus was used to perform neutralization assays in vitro. The flow cytometry results revealed that the infection efficiency of S pseudovirus was approximately 28.35 % (Fig. 3a). The pseudovirus luciferase assay results indicated that Nb91-hFc and Nb3-hFc neutralized SARS-CoV-2 pseudotyped virus with an IC_50_ value at approximately 54.07 nM (2.65 µg/mL) and 32.36 nM (1.79 µg/mL), respectively, whereas Nb82-hFc and Nb35-hFc had no inhibition effect (Fig. 3b). Similar results were obtained from fluorescence and flow cytometry assays (Fig. 3c).

### Detection the binding ability of multivalent neutralizing nanobodies

**Fig. 4:**
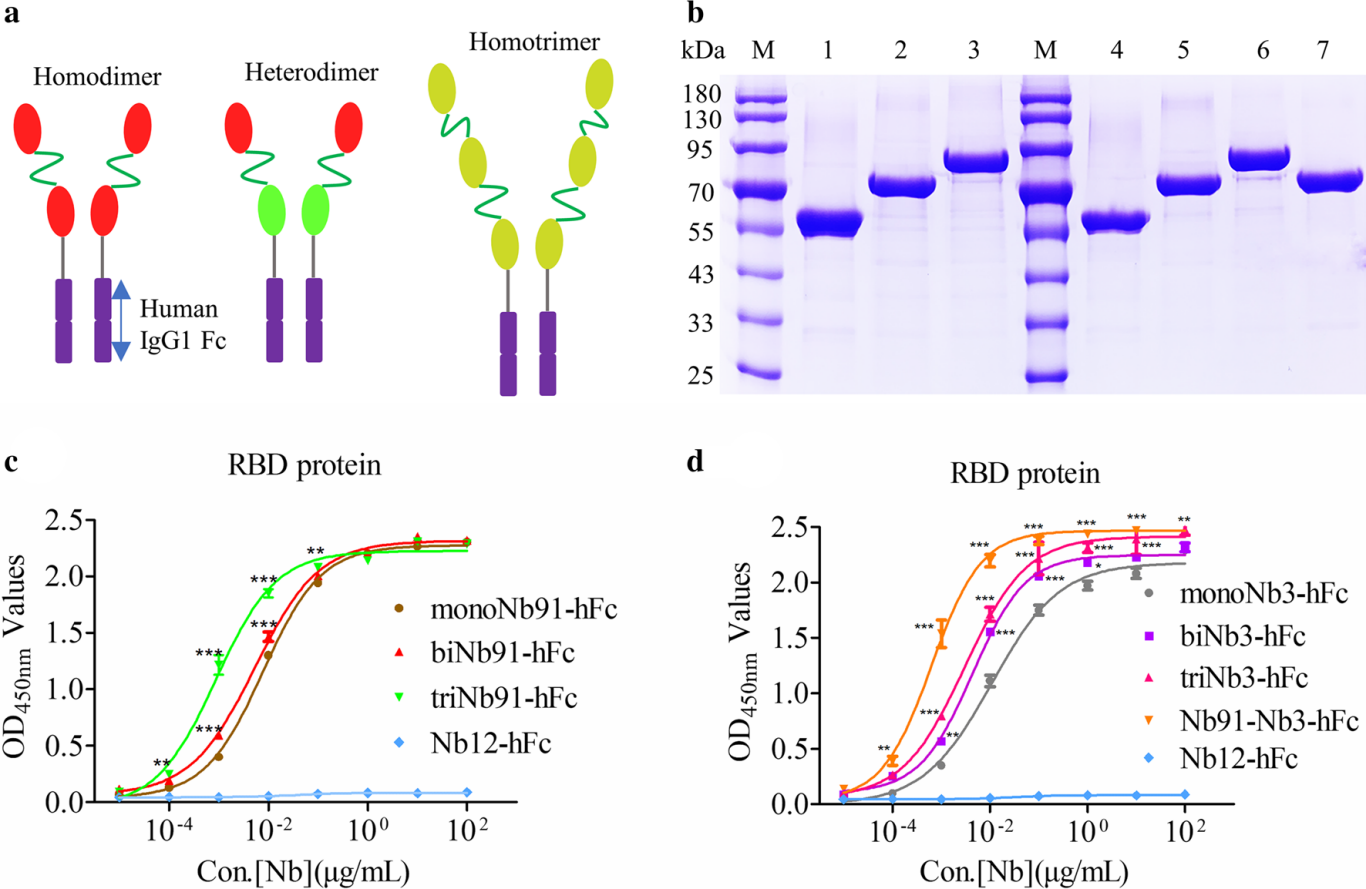
Development of multivalent nanobodies for highly efficient pseudovirus neutralization. **a** A schematic view of the multivalent nanobodies design. Homodimer, heterodimer and homotrimer were tandem linked with tail-to-head mode by flexibility linker. **b** The expression and purification of multivalent nanobodies fusions were analyzed with SDS-PAGE. M: Marker; lane 1 to 7: monoNb91-hFc, biNb91-hFc, triNb91-hFc, monoNb3-hFc, biNb3-hFc, triNb3-hFc and Nb91-Nb3-hFc, respectively. **c** and **d** Determination the binding affinity of multivalent nanobodies and monovalent Nbs with RBD protein using indirect ELISA

To further improve the antiviral properties of the neutralizing nanobodies against SARS-CoV-2, multivalent nanobodies were produced. Firstly, homodimer (biNb91-hFc, biNb3-hFc), heterodimer (Nb91-Nb3-hFc) and homotrimer (triNb91-hFc, triNb3-hFc) of Nb91 and Nb3 were tandem linked with flexible linker and constructed into pcDNA3.1-MCS-hFc vector (Fig. 4a). The expression procedure of multivalent nanobodies was consistent with monovalent Nbs. After purification, bivalent and trivalent VHHs were obtained from the supernatant of HEK293FT cells (Fig. 4b). The indirect ELISA results indicated that the binding affinity of homodimer and homotrimer with RBD protein were significantly higher than monovalent Nbs (*P* < 0.001) (Fig. 4c and d), and among them, Nb91-Nb3-hFc shows the highest affinity (*P* < 0.001) (Fig. 4d).

### Neutralization efficiency of the monovalent, bivalent and trivalent nanobodies

**Fig. 5:**
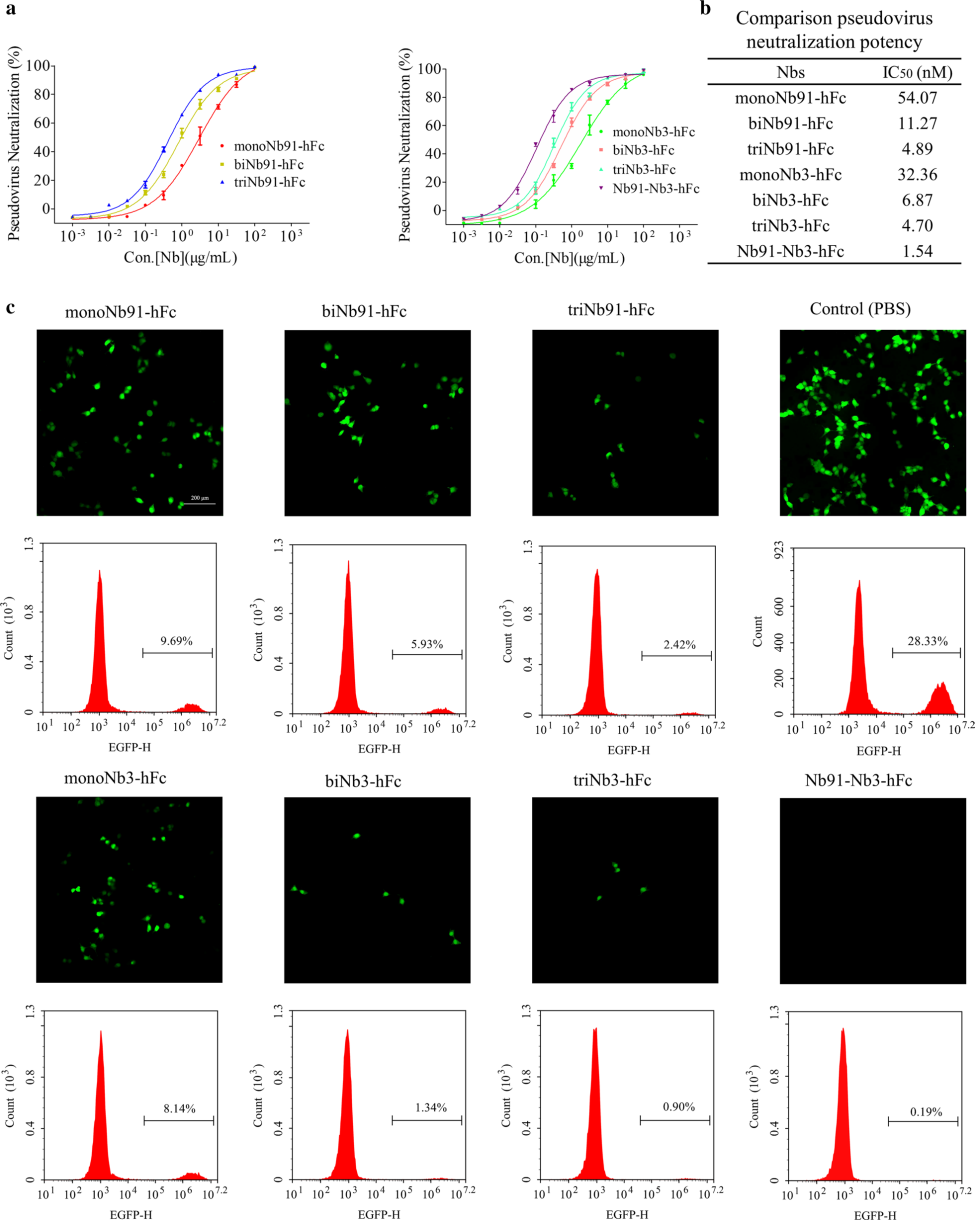
Evaluation the neutralizing abilities of the potent neutralizing nanobodies. **a** Neutralization potency was measured by using a pseudotyped virus luciferase neutralization assay. **b** A table summary of pseudotyped neutralization potency for seven nanobodies. IC_50_ were calculated by fitting a four-parameter logistic curve using Graphpad 5.0. **c** Neutralization potency of different multivalent nanobodies was performed using fluorescent microscopy and flow cytometry assays

The procedure of multivalent nanobodies pseduovirus neutralization assay were same with monovalent nanobodies. Results showed that the heterodimer nanobody Nb91-Nb3-hFc exhibited the highest neutralizing ability, with an IC_50_ at 1.54 nM against pseudotyped SARS-CoV-2 (Fig. 5a, b). The neutralizing ability of triNb91-hFc (IC_50_ = 4.89 nM) improved 11.06 fold compared to the monovalent form (IC_50_ = 54.07 nM), and triNb3-hFc (IC_50_ = 4.70 nM) improved 6.88 fold than monovalent construct (IC_50_ = 32.36 nM) by the pseudovius luciferase assay (Fig. 5a, b). These results revealed that the neutralizing ability of nanobodies could be obviously improved by tandem linking monovalent Nbs (Fig. 5b). Meanwhile, the antiviral activity of multivalent nanobodies were also determined using fluorescent microscopy and flow cytometry assays and similar results were obtained (Fig. 5c).

## Discussion

The COVID-19 pandemic has resulted in an unprecedented world public health crisis. Currently, efficient therapeutics for treatment of COVID-19 are lacking, and the development of a vaccine is likely to take at least 12–18 months [37, 38]. Convalescent plasma is utilized to improve therapeutic efficacy in patients with severe COVID-19 because the presence of neutralizing antibodies in the plasma of convalescent patients. Hence, neutralizing antibodies are promising for prevention and therapy of SARS-CoV-2 infection [39, 40]. Although the importance of neutralizing antibodies for protection is indisputable, cross-reactive antibodies or sub-optimal concentration of antibodies can promote pathology, resulting in a phenomenon known as antibody-dependent enhancement (ADE), which has been reported following secondary infections or vaccination with dengue virus and other viruses [41]. According to previous studies, the ADE mechanisms of SARS-CoV and MERS-CoV vaccines are mediated mainly by the engagement of Fc receptors (FcRs) expressed on macrophages, B cells and monocytes [42, 43]. So far, the clinical evidence has not shown the COVID-19 vaccines or antibodies to have such an effect. However, given ADE is a theoretical possibility, close monitoring is important to ensure that ADE can be ruled out as a side effect of COVID-19 vaccines or neutralizing antibodies.

To date, some monoclonal antibodies derived from COVID-19 patients, hybridoma and camelid VHH have been reported with various neutralizing efficiency [44–46]. Here, we isolated several nanobodies binding to spike protein and its RBD domain from a naïve VHH library. Previously, it is reported that multivalent nanobodies formed by tandem linking exhibited stronger binding affinity compare with the monomer nanobody [39, 47]. Consistently, in the present study, we also observed this phenomenon and found the heterodimer nanobody Nb91-Nb3-hFc showed the highest binding affinity and neutralizing activity. Our study indicated that naïve VHH library can be used as a potential resource for rapid acquisition and exploitation of antiviral nanobodies.

Since natural SARS-CoV-2 virus culture and assays must be carried out in a biosafety level-3 laboratory. Therefore, the antiviral activity of nanobodies against authentic viruses in vivo were not performed in the present study. However, compared with the natural viruses, pseudoviruses are well suited for virus entry assays because the pseudotyped particles have similar patterners, and can also efficiently simulate the infection process of natural viruses.

Compared with classical monoclonal antibodies, nanobodies show better tissue penetration and extravasation based on the advantage of small size. Recently, caplacizumab, a bivalent nanobody, as the first nanobody drug was approved by the FDA and European Medicines Agency (EMA) for treatment of patients with acquired thrombotic thrombocytopenic purpura [48–50]. Additionally, the lack of post-translational modifications of nanobodies allows it can be expressed in a variety of microbial systems including *Escherichia coli*, *Pichia pastoris* and *Saccharomyces cerevisiae*, which reducing production costs [51, 52]. However, the circulation half-life of nanobodies is significantly shorter in vivo due to their small size, which can be a limitation for disease treatment. Several approaches can be used to extend the half-life of nanobodies in vivo efficiently, such as PEGylation, fusion with Fc, HSA or HSA binding domain. On the other hand, for COVID-19 management, nanobodies might be delivered via nasal spray, and in this condition the half-life will be not a problem. For example, ALX-0171, a trivalent nanobody, can reduce the viral load in children with respiratory syncytial virus infection through aerosolized inhalation [53, 54]. Therefore, the neutralizing nanobodies identified in the present study may be exploited to develop aerosolized inhalation products for prevention of COVID-19.

## Conclusions

In the present work, four specific nanobodies against SARS-CoV-2-S or -RBD protein were screened from a non-immunized Bactrian camel VHH library. Nb91-hFc and Nb3-hFc exhibited highest affinity with RBD protein and neutralizing ability against S pseudovirus. We further compared the neutralizing capacities of tandem linked homo- or hetero-multivalent nanobodies. The results indicated that Nb91-Nb3-hFc possess the most potent neutralizing ability and may serve as an antiviral agent for prevention and treatment of COVID-19.

## Data Availability

All data generated or analyzed during this study are included in the article.
